# Metal–Organic Frameworks as Active Materials in Electronic Sensor Devices

**DOI:** 10.3390/s17051108

**Published:** 2017-05-12

**Authors:** Michael G. Campbell, Mircea Dincă

**Affiliations:** 1 Department of Chemistry, Barnard College, 3009 Broadway, New York, NY 10027, USA; 2 Department of Chemistry, Massachusetts Institute of Technology, 77 Massachusetts Avenue, Cambridge, MA 02139, USA

**Keywords:** metal–organic frameworks, porous materials, gas sensors, ion sensors, biosensors

## Abstract

In the past decade, advances in electrically conductive metal–organic frameworks (MOFs) and MOF-based electronic devices have created new opportunities for the development of next-generation sensors. Here we review this rapidly-growing field, with a focus on the different types of device configurations that have allowed for the use of MOFs as active components of electronic sensor devices.

## 1. Introduction

Owing to their high surface areas and robust chemical tunability based on a “bottom-up” synthetic approach, metal–organic frameworks (MOFs) are enabling new applications in chemical sensing [1,2,3]. However, because most MOFs have intrinsically low electrical conductivity, their use in electronic sensor devices is limited [4,5] and most reports in this area focus on optical responses (such as luminescence quenching or enhancement) [6,7,8,9,10,11], or more complicated device architectures such as MOF-coated microcantilevers [12] or quartz crystal microbalances [13,14,15]. Advances over the past decade have led to new synthetic approaches towards MOFs that simultaneously display permanent porosity and high electrical conductivity and/or charge mobility [16]. These advances are now enabling a new generation of MOF-based electronic sensor devices, which display great promise as platform for the development of improved sensing technologies.

In this Review, we survey the burgeoning field of MOF-based electronic devices for chemical sensing, focusing on the different types of devices and measurement techniques that have been used to date. We will examine here only sensor devices in which the MOF functions as an active electrical component; therefore, we exclude devices in which the MOF plays a passive role such as an adsorbent coating or a selective molecular sieve [17,18].

## 2. MOF-Based Gas Sensors 

To date, the majority of the published studies on MOF-based sensor devices have looked at sensing of gases and chemical vapors. A variety of device types and configurations have been investigated, in large part as an effort to overcome the low conductivity of most MOF materials that have been tested. Despite the limitations imposed by low conductivity, significant progress has been made in recent years, and MOF-based electronic sensor devices are poised to make a meaningful impact on gas sensing.

### 2.1. Impedance Sensors

**Figure 1:**
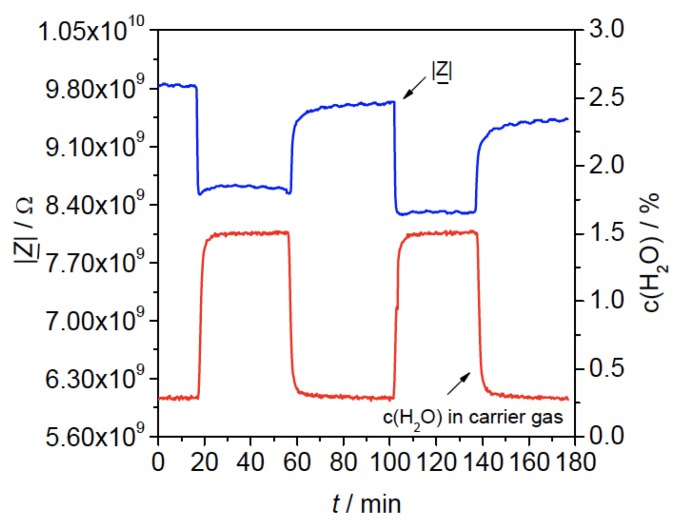
Impedance sensing response (|Z|), measured at 1 Hz, of a Fe-BTC thick film sensor device to two sequential exposures to 1.5% (*v*/*v*) H_2_O/N_2_. Image reproduced with permission from reference [19], copyright 2008 by the authors.

The first successful examples of MOF-based electronic sensor devices used impedance spectroscopy to look at responses to different gases and vapors; in this measurement technique, a high bulk electrical conductivity is not required for device performance. In 2009, Achmann et al. investigated five materials: Al-BDC [Al(OH)(BDC)], Fe-BTC [Fe^III^(BTC)], Cu-BTC [Cu_3_(BTC)_2_], Li-doped Fe-BTC, and Fe(II)-doped Fe-BTC (BDC = 1,4-benzenedicarboxylate; BTC = 1,3,5-benzenetricarboxylate) [19]. Two device configurations were used, thick films coated onto interdigitated electrodes (IDEs) and pressed pellets. Changes in impedance were measured with varying concentrations of O_2_, CO_2_, C_3_H_8_, NO, H_2_, ethanol, and methanol, over a range of temperature (120–240 °C) and humidity levels. Of the materials tested, only Fe-BTC displayed any observable responses, to ethanol, methanol, and H_2_O, with the response toward water being the strongest (Figure 1).

In a related study published in 2013, Zhang et al. fabricated devices on IDEs using NH_2_-MIL-125(Ti) ([Ti_8_O_8_(OH)_4_(abdc)_6_]; abdc = 2-amino-1,4-benzenedicarboxylate) [20]. These devices were sensitive to changes in relative humidity in the range 11–95%, at an optimized frequency of 100 Hz, with response and recovery times of <1 min.

### 2.2. Chemicapacitive Sensors

Changes in capacitance have also been investigated for MOF-based sensor devices. In 2016, Hosseini et al. reported the fabrication of a capacitive senor using Cu-BTC, which was grown directly onto a copper substrate using electrochemical synthesis [21]. In this case, the copper substrate for MOF film growth served as the back electrode, and the top electrode was made using connected spots of Ag paste on top of the Cu-BTC film. The devices displayed a reversible “turn-on” response in capacitance in response to both ethanol and methanol vapor, with response/recovery times on the order of several minutes. A much weaker “turn-off” response was observed for non-polar vapors such as *n*-hexane.

**Figure 2:**
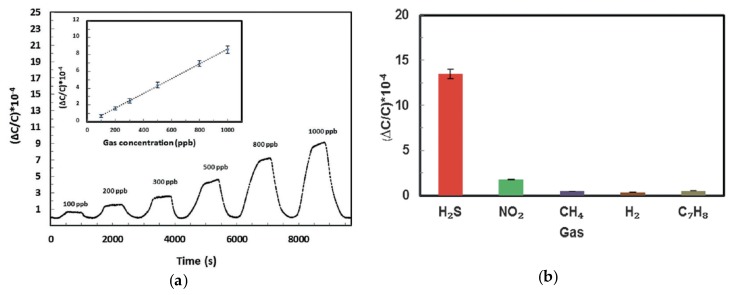
Chemicapacitive sensing of H_2_S using a fumarate-based metal–organic frameworks (MOF) grown onto interdigitated electrodes (IDEs): (**a**) Detection of H_2_S at ppb levels, showing a linear dependence on concentration; (**b**) Demonstration of selectivity compared to other gases. Images reproduced with permission from reference [22], copyright 2016 Wiley-VCH Verlag GmbH & Co. KGaA, Weinheim.

Later in 2016, Yassine et al. reported a capacitive sensor fabricated by growing thin films of a fumarate—based yttrium MOF, in which hexanuclear Y clusters are connected by fumarate ligands in a UiO-66-type structure, onto IDEs that had been functionalized with a self-assembled monolayer of 11-mercaptoundecanol [22]. These capacitive sensors displayed extraordinary sensitivity to H_2_S vapors (≤100 ppb) at room temperature, and high selectivity for H_2_S over other gases such as NO_2_, CH_4_, H_2_, and toluene (Figure 2). The devices showed linear changes in capacitance over several orders of magnitude H_2_S concentration (from 100 ppb to 100 ppm) and were stable up to 12 weeks.

In addition to the use of neat MOFs as the active component in capacitive sensors, composites of MOFs with organic polymers have also been investigated as active materials. In 2016, Sachdeva et al. reported the use of a NH_2_-MIL-53(Al)/polyimide composite to fabricate a capacitive sensor for methanol vapor [23]. Devices fabricated with the composite material displayed a lower detection limit as compared to the polymer alone or bare electrodes. The use of MOF-based hybrid materials for sensing has not yet been extensively explored, and may provide exciting new opportunities for materials development.

### 2.3. Chemiresistive Sensors

Chemiresistive sensors are perhaps the simplest and therefore most desirable sensor devices, and have been extensively studied for other porous nanomaterials such as carbon nanotubes (CNTs) [24,25]. Use of MOFs as the active material in chemiresistive sensors has only recently been investigated, in large part due to the lack of MOFs with suitable electrical conductivity. The development of new synthetic approaches toward MOFs that display both porosity and electrical conductivity has enabled the first applications of these materials in chemiresistive sensors.

**Figure 3:**
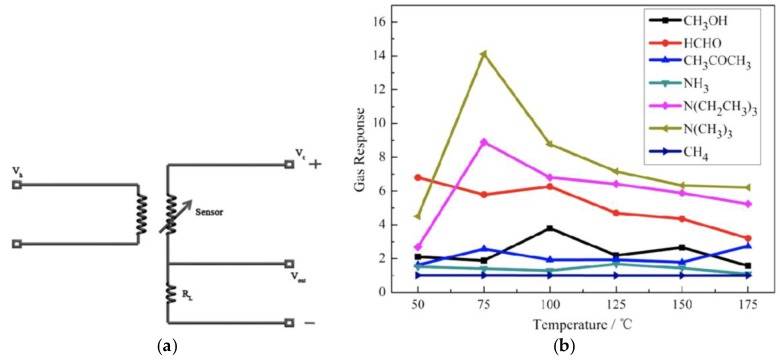
Chemical vapor sensing with a Co(im)_2_ sensor: (**a**) Schematic of the MOF sensor device (V_h_ = heating voltage; V_c_ = circuit voltage; V_out_ = output voltage; R_L_ = load resistance); (**b**) Response (defined as the ratio of device resistance under the vapor atmosphere versus under air) of the sensor to various chemical vapors as a function of temperature (100 ppm vapor concentrations, exposure times ~30 min). Images reproduced with permission from reference [27], copyright 2014 American Chemical Society.

In 2014, the Zhang group reported two examples of using zeolitic imidizolate frameworks (ZIFs) in resistive sensors, by coating ZIFs onto IDEs followed by annealing. It was first reported that Co(mim)_2_ (ZIF-67; mim = 2-methylimidizolate) could be used to detect formaldehyde vapor, at concentrations as low as 5 ppm [26]. However, the sensor required elevated temperatures (150 °C) to operate, and the response/recovery times were on the order of several minutes over a concentration range of 5–500 ppm formaldehyde. Subsequently, the related material Co(im)_2_ (im = imidazolate) was used for the detection of trimethylamine vapor [27]. The devices displayed promising long-term stability, over the course of several weeks, but again elevated temperatures (75 °C) were required for operation and long response/recovery times were observed (Figure 3). The issues of temperature and response times for the reported ZIF-based sensors are likely a result of the low intrinsic conductivity of Co ZIF materials.

MOFs with much higher electrical conductivity have appeared beginning in 2012, with the first reports of layered two-dimensional (2D) π-conjugated MOFs [28,29,30]. These advances enabled the first examples of MOF-based chemiresistors that could operate at room temperature and with minimal power requirements. In 2015, Campbell et al. demonstrated that the conductive MOF Cu_3_(HITP)_2_ (HITP = 2,3,6,7,10,11-hexaiminotriphenylene) could be used to fabricate chemiresistive sensors with sub-ppm sensitivity to ammonia vapor, at room temperature and with an applied potential of 100 mV [31]. Device performance was maintained under air with up to 60% relative humidity. It was also found that devices fabricated from the isostructural MOF Ni_3_(HITP)_2_ did not display sensitivity to NH_3_ vapor under identical conditions, indicating the potential for tuning the sensor’s response based on the MOF’s chemical structure.

**Figure 4:**
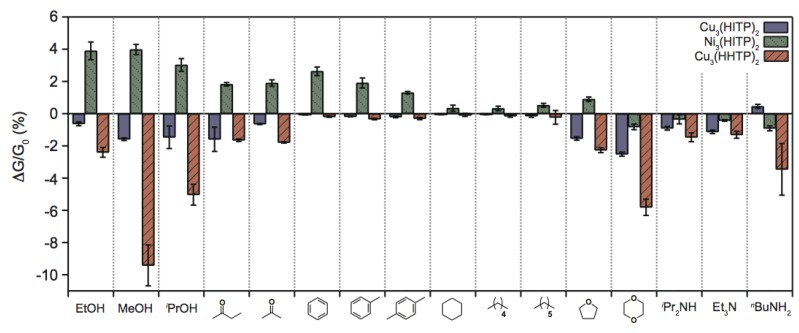
Responses of a chemiresistive sensor array, constructed from conductive 2D MOFs, to different categories of volatile organic compounds (VOCs), where ∆G/G_0_ is the relative response (change in conductance) upon a 30 s exposure to 200 ppm of the VOC vapor at room temperature; each response is averaged from 12 measurements (4 exposures to 3 separate devices for each MOF); error bars show one standard deviation. Image adapted with permission from reference [32], copyright 2015 American Chemical Society.

Subsequently, Campbell et al. reported the use of structurally-related 2D MOFs to construct a cross-reactive sensor array that could successfully discriminate between several classes of volatile organic compounds (VOCs) based on functional group [32]. The MOFs Ni_3_(HITP)_2_, Cu_3_(HITP)_2_, and Cu_3_(HHTP)_2_ (HHTP = 2,3,6,7,10,11-hexahydroxytriphenylene) were used, and the chemiresistive responses of the devices were measured towards various VOC vapors at 200 ppm concentration levels (Figure 4). The individual MOF components of the sensor array displayed differential responses to various groups of chemicals. Using statistical analysis, it was shown that the MOFs’ chemiresistive responses could be used to distinguish between categories of VOCs with >90% accuracy. Additionally, concentration-dependent studies with amine vapors indicated that multiple sensing mechanisms are operative, with high degrees of orthogonality. The mechanisms of VOC sensing with conductive 2D MOFs are still under investigation, but the preliminary results reported to date suggest that MOF-based chemiresistors are a promising platform that may offer advantages over existing technologies.

In 2016, Smith et al. reported the direct growth of M_3_(HHTP)_2_ films (M = Cu, Ni) onto graphitic electrodes that had been patterned onto polymer films [33]. Consistent with the previously-reported studies, devices fabricated in this manner from Cu_3_(HHTP)_2_ displayed a chemiresistive response towards NH_3_ vapor, while devices fabricated from Ni_3_(HHTP)_2_ did not display an observable response. The authors further showed that the M_3_(HHTP)_2_ devices could be used to sense NO and H_2_S, and that a sensor array using the two types of devices could successfully differentiate between H_2_O, NH_3_, NO, and H_2_S vapors. These results reinforce the potential for creating simple MOF-based chemiresistor devices and arrays that can offer high selectivity in gas sensing applications.

It is noteworthy that, in the work reported to date on MOF-based chemiresistors, a wide range of device fabrication methods have been used: coating the MOFs onto electrodes using a solvated “paste” [26]; drop-casting [31,32]; solvent-free mechanical abrasion [32]; and in situ film growth [33]. These studies demonstrate that multiple fabrication techniques are viable; however, very little work has been done so far on understanding the effect of different deposition methods or film morphologies on sensing performance. In moving towards practical applications, these additional technical issues will need to be systematically investigated.

### 2.4. Kelvin Probe and Field Effect Transistor Sensors

**Figure 5:**
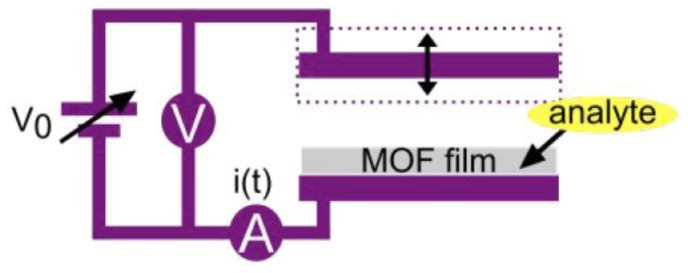
Schematic drawing of the Kelvin probe setup for measuring changes in work function: the MOF film is grown or deposited on top of a stationary electrode, which is connected to an oscillating reference electrode, with both electrodes exposed to the analyte vapor. Image reproduced with permission from reference [42], copyright 2016 The Royal Society of Chemistry.

As with chemiresistors, field effect transistors (FETs) are a useful device configuration for sensing because they have the potential to be sensitive, robust, and compatible with inexpensive and scalable fabrication techniques [34,35]. Examples of MOF-based FETs are rare thus far [36,37], and to our knowledge have not been used for chemical vapor sensing. A common mechanism for FET-based sensor devices is a change in work function of the active material upon exposure to the analyte vapor. Changes in work function can also be measured using the Kelvin probe technique, which is compatible with MOFs that do not exhibit sufficient electrical conductivity for use in FETs (Figure 5). Several groups have investigated vapor sensing with MOFs via changes in work function, using the Kelvin probe method. Although such a device configuration is not practical for use in sensing technologies, these preliminary studies provide evidence that work function gas sensors such as MOF-based FETs should display good performance if they can be experimentally realized.

The first example of this approach was reported by Pohle et al. In 2011, using Cu-BTC [38]. Exposures to a variety of gases (hexanal, pentanal, toluene, dimethyl ether, ammonia, H_2_S, ethanol, acetone) were studied over a temperature range of 25–200 °C. Temperature had a significant effect on performance, with higher temperatures leading to a stronger response. Although almost all of the polar analytes produced an observable response, size-exclusion effects were also observed: pentanal produced a response whereas hexanal did not; additionally, NH_3_ and H_2_S produced a response whereas dimethyl ether did not. Finally, the metal used for the back electrode of the device (Au or Pt) had a pronounced effect on response, suggesting that the observed responses result from a combined effect of the MOF and the metal substrate on which it was deposited. A follow-up study in 2013 by Davydovskaya et al. further showed the effect of analyte size on performance for aldehyde sensing with Cu-BTC [39]. As in the initial report, studies comparing pentanal and hexanal showed that only pentanal produced a response, consistent with a size-exclusion effect; however, additional measurements with ethanal and propanal showed that these smaller aldehydes also produced a much weaker response as compared to pentanal. Furthermore, the response to pentanal could only be observed at relative humidity levels ≥30%, indicating that water vapor also plays a role in aldehyde sensing. Overall, these studies point to the complexity of MOF-analyte interactions that can occur in sensing: the ability to tune pore size and chemical functionality within the MOF is a benefit for sensor development, but these complexities can also make elucidation of sensing mechanisms challenging.

Further work by Davydovskaya et al. in 2014 examined the sensing performance for a series of M-BTC frameworks (M = Co, Ni, Cd, Al) upon exposure to various linear alkanes and linear aliphatic alcohols [40]. As expected, non-polar alkanes had a negligible effect on work function for all of the MOFs studied, whereas the polar alcohols produced changes in work function. Alcohols with longer carbon chains were shown to produce a stronger response, and humidity level was again shown to have an impact on response. Surprisingly, the sensing performance was not significantly impacted by the identity of the MOF’s metal center, with all of the MOFs showing comparable changes in work function. A related study by Pentyala et al. in 2016 also showed similar alcohol sensing behavior for Zn-BTC [41]. As with the initial studies reported in 2011, these results may suggest that in these devices the MOF/substrate interaction is more important for sensing than the electronic structure of the MOF itself. 

**Figure 6:**
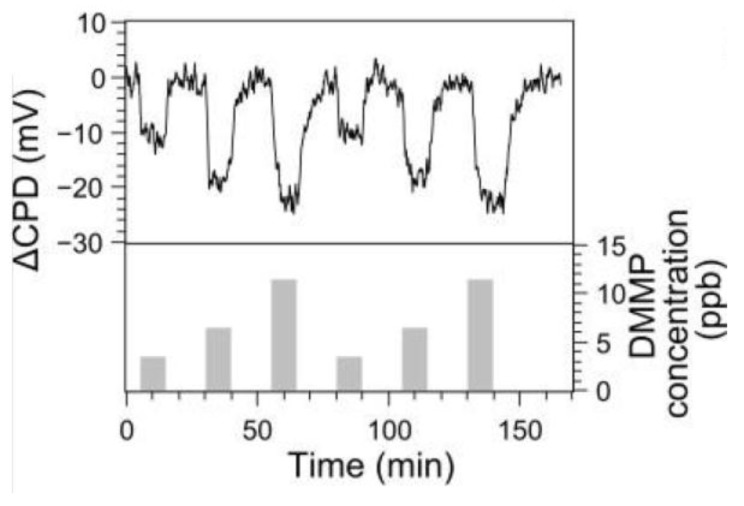
Sensing response of UiO-66-NH_2_ to dimethyl methylphosphonate (DMMP) at ppb concentration levels, measured using a Kelvin probe setup (CPD = contact potential difference). Image reproduced with permission from reference [42], copyright 2016 The Royal Society of Chemistry.

As the most recent example in this class, in 2016 Stassen et al. applied the Kelvin probe approach to the detection of alkyl phosphonate nerve agents using UiO-66-NH_2_, wherein [Zr_6_O_4_(OH)_4_]^12+^ clusters are connected by 2-amino-1,4-benzenedicarboxylate ligands [42]. The test molecule dimethyl methylphosphonate (DMMP), used as a mimic for nerve agents such as Sarin gas, could be reversibly detected by changes in work function at concentrations as low as 3 ppb (Figure 6). Even under high-humidity conditions (50% relative humidity), ppb-level concentrations of DMMP could be detected, and a lower-limit of detection was calculated to be 2 ppb. A combination of experimental and theoretical studies suggests that the high sensitivity towards DMMP results from the presence of missing-linker defect sites that create unique binding pockets within the material.

## 3. MOF-Based Ion Sensors and Biosensors

There have been limited examples so far of using MOFs for solution-phase sensing of ions or biologically relevant molecules. The lack of work in this area may be due, in part, to the fact that many MOFs are not stable in water; however, water-stable MOFs have been extensively developed in recent years, including some of the conductive 2D MOFs described above. This area of research therefore seems ripe for exploration in the near future.

**Figure 7:**
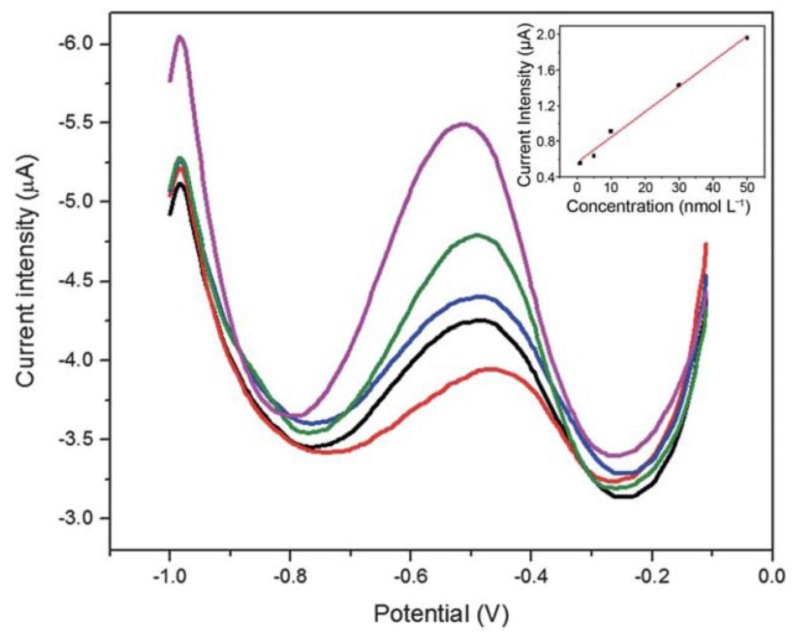
Sensing of lead in solution using an electrode modified with a Cu-BTC/CNT composite; the plot shows differential pulse voltammograms recorded at varying lead concentrations, and the inset shows the linear relationship between peak current and lead concentration. Image reproduced with permission from reference [43], copyright 2013 The Royal Society of Chemistry.

An example of ion sensing was reported in 2013 by Wang et al., in which Cu-BTC/CNT composites were used for the detection of nanomolar quantities of lead [43]. The experiment was conducted by modifying a glassy carbon electrode (GCE) surface with the MOF/CNT composite, itself produced solvothermally. Lead, from solution, was allowed to accumulate on the modified electrode surface, and was then quantified using differential pulse anodic stripping voltammetry (Figure 7). The modified electrodes were more sensitive towards lead as compared to a bare GCE surface, and the measurement method was validated by measuring several standard lead-containing water samples and comparing to the certified values for the standards.

Co(mim)_2_ (ZIF-67) has also been used to prepare modified GCEs, as reported by Zhao et al. in 2015 [44]. These ZIF-modified electrodes efficiently detected glutathione, a tripeptide that plays a key role in cellular processes, in solution. In addition to the chemical structure, it was found that the faceting of the ZIF-67 crystals had an impact on the sensing performance, with the {110} facets proving optimal. 

A handful of other examples of biosensing using MOFs have been reported, targeting glucose and lipopolysaccharide [45,46]. However, in these cases the MOFs serve primarily as carriers or encapsulating agents, rather than as the active electrocatalysts. Moving forward, we expect that recent developments in using water-stable, conductive MOFs as electocatalysts will lead to expanded applications in aqueous sensing of ions and biomolecules [47,48].

## 4. Outlook

The field of MOF-based sensors is rapidly expanding to include electronic sensor devices that feature MOFs as an active component. Although early work in this area was limited by a lack of efficient signal transduction due to the insulating behavior of most MOFs, the work described here clearly shows that the field is beginning to move beyond these limitations. Most significant in this sense is the continued development of chemical strategies for the synthesis of electrically conductive MOFs, which will provide a source of promising new materials candidates. Computational studies that identify potential candidates for devices from among the thousands of known MOFs will also prove important: such studies have already pointed towards possible materials for MOF-based sensors and sensor arrays [49,50,51]. Furthermore, successful demonstrations of MOF-based functional devices have begun to appear in recent years [52,53], including the sensor devices described here. We believe that a focus should be placed on targeting practical devices with the potential for “real-world” use, such as chemiresistors and FETs. Because of their ease of fabrication, low power requirements, and ready integration into more complex circuitry, these categories of devices represent an exciting opportunity for MOFs to make a significant impact in the field of sensing.
